# Persistence of duplicated PAC_1 _receptors in the teleost, *Sparus auratus*

**DOI:** 10.1186/1471-2148-7-221

**Published:** 2007-11-12

**Authors:** João CR Cardoso, Edwin CJM de Vet, Bruno Louro, Greg Elgar, Melody S Clark, Deborah M Power

**Affiliations:** 1 CCMAR, Molecular and Comparative Endocrinology, University of Algarve, 8005-139 Faro, Portugal; 2 Laboratory of Molecular Signalling, The Babraham Institute, CB2 4AT, Cambridge, UK; 3 School of Biological and Chemical Sciences, Queen Mary, University of London, Mile End Road, E1 4NS, London, UK; 4 British Antarctic Survey, Natural Environment Research Council, High Cross, Madingley Road, Cambridge, CB3 0ET, Cambridge, UK

## Abstract

**Background:**

Duplicated genes are common in vertebrate genomes. Their persistence is assumed to be either a consequence of gain of novel function (neofunctionalisation) or partitioning of the function of the ancestral molecule (sub-functionalisation). Surprisingly few studies have evaluated the extent of such modifications despite the numerous duplicated receptor and ligand genes identified in vertebrate genomes to date. In order to study the importance of function in the maintenance of duplicated genes, sea bream (*Sparus auratus*) PAC_1 _receptors, sequence homologues of the mammalian receptor specific for PACAP (Pituitary Adenylate Cyclase-Activating Polypeptide), were studied. These receptors belong to family 2 GPCRs and most of their members are duplicated in teleosts although the reason why both persist in the genome is unknown.

**Results:**

Duplicate sea bream PACAP receptor genes (sbPAC_1_A and sbPAC_1_B), members of family 2 GPCRs, were isolated and share 77% amino acid sequence identity. RT-PCR with specific primers for each gene revealed that they have a differential tissue distribution which overlaps with the distribution of the single mammalian receptor. Furthermore, in common with mammals, the teleost genes undergo alternative splicing and a PAC_1_Ahop1 isoform has been characterised. Duplicated orthologous receptors have also been identified in other teleost genomes and their distribution profile suggests that function may be species specific. Functional analysis of the paralogue sbPAC_1_s in Cos7 cells revealed that they are strongly stimulated in the presence of mammalian PACAP_27 _and PACAP_38 _and far less with VIP (Vasoactive Intestinal Peptide). The sbPAC_1 _receptors are equally stimulated (LOG_EC50 _values for maximal cAMP production) in the presence of PACAP_27 _(-8.74 ± 0.29 M and -9.15 ± 0.21 M, respectively for sbPAC_1_A and sbPAC_1_B, P > 0.05) and PACAP_38 _(-8.54 ± 0.18 M and -8.92 ± 0.24 M, respectively for sbPAC_1_A and sbPAC_1_B, P > 0.05). Human VIP was found to stimulate sbPAC_1_A (-7.23 ± 0.20 M) more strongly than sbPAC_1_B (-6.57 ± 0.14 M, P < 0.05) and human secretin (SCT), which has not so far been identified in fish genomes, caused negligible stimulation of both receptors.

**Conclusion:**

The existence of functionally divergent duplicate sbPAC_1 _receptors is in line with previously proposed theories about the origin and maintenance of duplicated genes. Sea bream PAC_1 _duplicate receptors resemble the typical mammalian PAC_1_, and PACAP peptides were found to be more effective than VIP in stimulating cAMP production, although sbPAC_1_A was more responsive for VIP than sbPAC_1_B. These results together with the highly divergent pattern of tissue distribution suggest that a process involving neofunctionalisation occurred after receptor duplication within the fish lineage and probably accounts for their persistence in the genome. The characterisation of further duplicated receptors and their ligands should provide insights into the evolution and function of novel protein-protein interactions associated with the vertebrate radiation.

## Background

Increased gene number and complexity are generally assumed to have contributed to the success of vertebrates. The evolutionary driving forces behind this are still under debate however gene and/or genome duplications and exon shuffling events are proposed to have been of fundamental importance [1-5]. The increased complexity of metazoan genomes have been attributed to rounds of gene or whole genome duplication [1,6-9]. Analysis of metazoa genomes reveals that a remarkable percentage of duplicated genes exist [10-13] and whilst some genes decay to non-functionality and are subsequently eliminated from the genome, others are maintained either through the acquisition of novel functions (neofunctionalisation) or by partitioning the function of the ancestral molecule between the duplicated isoforms (subfunctionalisation).

The secretin family of G-protein coupled receptors (GPCRs) (a.k.a. family 2 GPCRs) is a large hormone and neuropeptide receptor gene family present in metazoan genomes. Members of this family have been identified in both protostomes and deuterostomes [14-16] and their conserved sequence and gene organisation has led to the proposal that they evolved from a common ancestral gene as a consequence of total, or partial genome duplication [14]. Vasoactive Intestinal Peptide (VIP) and Pituitary Adenylate Cyclase Activating Polypeptide (PACAP) receptors (VPAC and PAC_1_, respectively) are closely related members of family 2 GPCRs. They are important pharmaceutical targets as their ligands, the brain-gut peptides VIP and PACAP, control a number of important physiological functions in mammals [17,18].

In humans three receptors exist, PAC_1_, VPAC_1 _and VPAC_2 _and binding studies reveal that VPACs are able to bind the ligands, VIP and PACAP with similar affinities, while PAC_1 _preferentially binds PACAP [18,19]. In vertebrates, activation of PAC_1_/VPAC receptors occurs via three intracellular transduction mechanisms. These involve either cyclic AMP (cAMP) production, IP turnover via PLC or/and calcium mobilization as a consequence of the activation of a G-protein complex [18,20-22]. In mammals, five PAC_1 _isoforms, which result from the insertion of one or two, 28 (hip or hop1 variant) or 27 (hop2 variant) amino acid cassettes in intracellular loop 3 (IL3) have been identified [23,24]. In addition, two VPAC_1 _and VPAC_2 _isoforms have been recently described which lack TM6 and TM7 [25]. In other vertebrates PAC_1 _splice isoforms have been isolated, however no alternative splice forms of VPAC have yet been identified and in vertebrates these latter receptors do not activate the IP3 signalling pathway [26-28].

Teleosts, which diverged from the tetrapod lineage approximately 450 million years ago (MYA), represent one of the most successful vertebrate groups with over 25,000 species. The existence of a variety of teleost genome sizes and ploidy levels has made them very useful for studies of gene evolution and function [29]. Recently duplicated PAC_1 _and VPAC receptor genes have been identified in teleosts using *in silico *approaches [16] and in *Takifugu *they have a differential tissue distribution [15]. In the present study duplicate PAC_1 _cDNAs were isolated from the marine teleost, sea bream (*Sparus auratus*) and their tissue expression and functional profile characterised using the peptides VIP, PACAP_27_, PACAP_38 _and SCT (secretin). The persistence of duplicate sbPAC_1 _receptors in teleost genomes is discussed in relation to the current proposed theories for gene evolution in vertebrates.

## Results

### Sea bream duplicate PAC_1 _receptors

**Figure 1:**
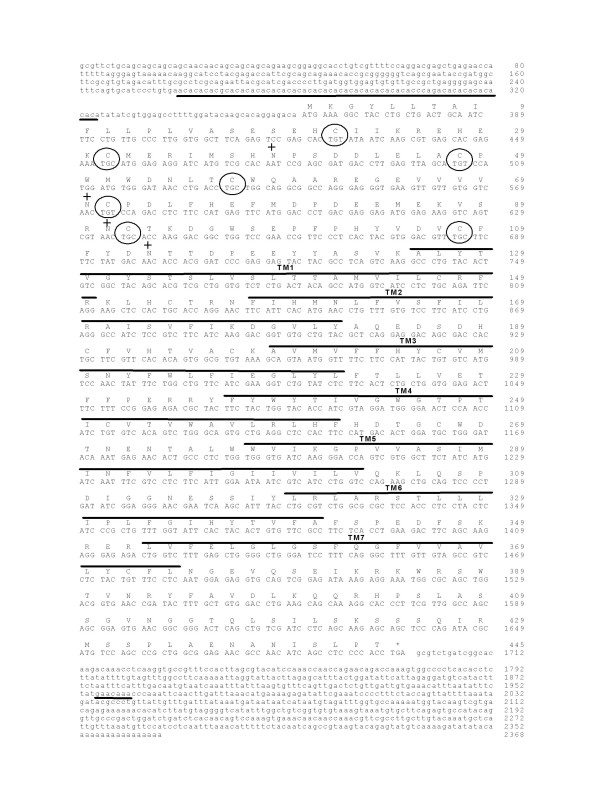
**Nucleotide and predicted protein sequence of sbPAC_1_A receptor**. Seven TM domains are highlighted with black lines and the conserved N-terminal cysteine residues and putative N-glycosylation sites are annotated respectively by a circle and by "+". The microsatellite identified in the 5'UTR is underlined and the stop codon is indicated by "*".

**Figure 2:**
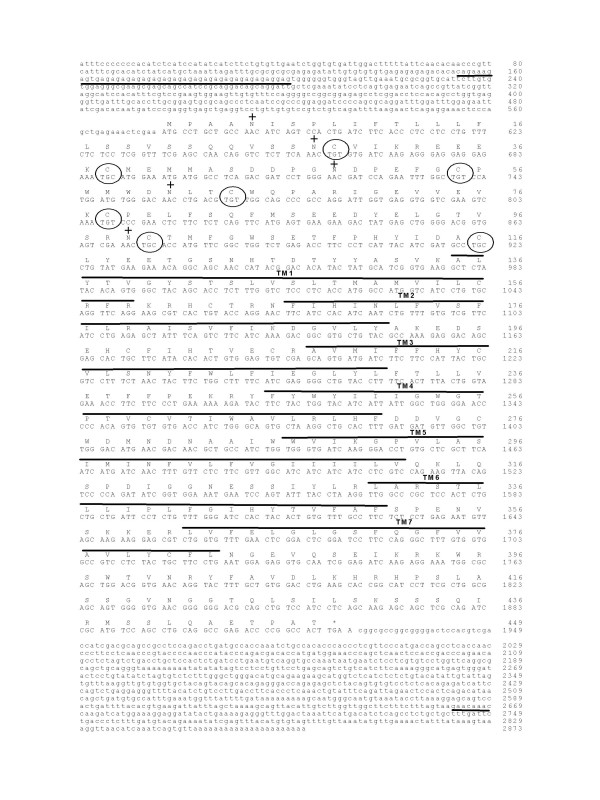
**Nucleotide and predicted protein sequence of sbPAC_1_B receptor**. The seven TM domains are highlighted with black lines and the conserved N-terminal cysteine residues and putative N-glycosylation sites are annotated respectively by a circle and by "+". The microsatellite identified in the 5'UTR is underlined and stop codon is indicated by "*".

Two complete putative sbPAC_1 _cDNAs, namely sbPK713 (2368 bp) and sbPP1C (2791 bp), were isolated from a sea bream kidney and pituitary cDNA libraries, respectively (Figures 1 and 2). The isolated cDNAs encode putative proteins of 444 and 448 amino acids respectively, and share an overall amino acid identity of 77% due to the highly conserved TM domains and C-terminal regions; the N-terminal domain is only 48% identical. The sbPK713 and sbPP1C cDNAs share 91% and 86% amino acid sequence identity respectively, with the previously identified *Takifugu *PAC_1_A and PAC_1_B and for this reason were designated sbPAC_1_A (AJ514930) and sbPAC_1_B (AJ514931). Analysis of sea bream genomic DNA by RT-PCR coupled with Southern blot (data not shown) confirmed that both sea bream PAC_1 _cDNAs are encoded by single genes and are not the result of alternative splicing. This observation is confirmed by mapping of sbPAC_1_A and sbPAC_1_B genes in the recently released sea bream gene-based radiation hybrid (RH) map, to group 6 (RH6) and 2 (RH2), respectively [30].

A microsatellite sequence is present in the 5'UTR region of both receptors and sbPAC_1_A contains an imperfect (AC)_32 _dinucleotide repeat and sbPAC_1_B an imperfect (GA)_28 _repeat upstream of the initiation codon (Figures 1 and 2). Genotyping of the microsatellites using genomic DNA from sea bream caught at different geographic locations and a family panel, revealed both microsatellites are polymorphic and the locus sbPAC_1_B scores more alleles (8 alleles) than sbPAC_1_A (4 alleles) (Additional file 1).

### Sequence comparisons and phylogenetic analysis

**Figure 3:**
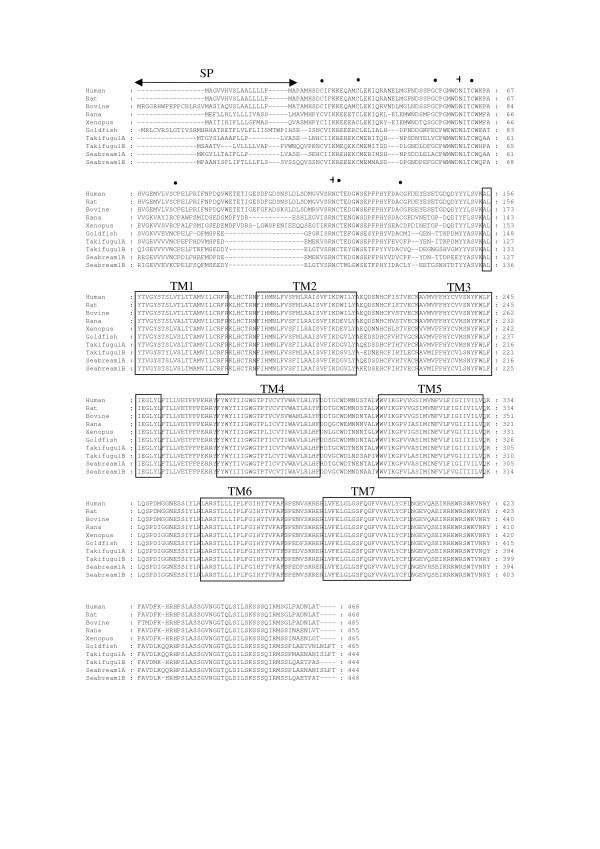
**Multiple sequence alignment of vertebrate PAC_1 _receptors**. The seven TM domains are boxed, the P-D-I/M motif indicated by "○"and the R-L-A-R motif by "*". The alternative receptor hop splice isoform is indicated by a dotted box and the putative signal peptide sequence with a double ended arrow. Conserved cysteines and putative N-glycosylation sites in the N-terminal domain are indicated by "●" and "+", respectively. Accession numbers of sequences used in the multiple amino acid sequence alignment were; human (*Homo sapiens*, P41586), rat (*Rattus norvegicus*, P32215), bovine (*Bos taurus*, Q29627), Rana (*Rana ridibunda*, Q90Y07), Xenopus (*Xenopus laevis*, Q9PTK1), Goldfish (*Carassius auratus*, O7376, Takifugu (*Takifugu rubripes*, AJ494861 for PAC_1_A and AJ490804 for PAC_1_B) and sea bream sbPAC_1_A (AJ514930) and sbPAC_1_B (AJ514931).

Sequence comparison of sbPAC_1_A and sbPAC_1_B with vertebrate homologue receptors revealed six conserved cysteine residues and two putative N-glycosylation sites at the N-terminal domain which are implicated in ligand-binding [20](Figure 3). Additional conserved motifs characteristic of PAC_1 _are also present. Within intracellular loop three (IL3) the amino acid motifs, P-D-M and R-L-A-R (Basic-L/A-L/A/V/S-Basic), important for functional coupling with the Gsα protein, are conserved in the teleost and tetrapod receptors. Amino acid residues K^322 ^and E^394^, which are involved in activation of the cAMP pathway in humans [21] are also conserved suggesting that a similar receptor signalling pathway may exists in sea bream. A putative signal peptide sequence is also present along with the consensus signature motif of TM7 in mammalian PACAP and VIP receptors: FQGBBVXXBYCFXNXEVXQ (where X is any amino acid and B is a basic amino acid [15,31]. Duplicate PAC_1 _genes also exist in other teleosts genomes, such as stickleback (ENSGACP00000022696 and ENSGACP00000007143), *Tetraodon *(GSTENP00011829001 and GSTENP00034495001), Medaka (ENSORLP00000022250 and ENSORLP00000016315) and zebrafish (NM_001013444 and XM_701685) (Table 1). In contrast, a single copy of a PAC_1 _gene was identified in the Atlantic salmon (*Salmon salar*, CK885244) and rainbow trout (*Oncorhynchus mykiss*, AY706216). It seems likely that further PAC_1 _receptors exist in salmonids and it is expected that these will be identified as sequence coverage of their genomes is enriched.

**Table 1:** Accession numbers of the putative teleost PAC_1_, VPAC, PRPR and GHRHR identified *in silico*.

	***PAC1***	***VPAC***_1_	***VPAC***_2_	***PRPR***	***GHRHR***
***TAKIFUGU***	AJ494861	AJ296144	AJ408877	AJ296145	SINFRUP00000146089
	AJ490804	AJ296143	AJ408878	SINFRUP00000161807	
***TETRAODON***	GSTENP00011829001	GSTENP00023906001	GSTENP00016553001	GSTENP00034494001	GSTENT00012645001
	GSTENP00034495001	GSTENP00015127001		GSTENP00011830001	
***ZEBRAFISH***	NM_001013444	NM_001013353	Not identified	ENSDARP00000054330	DQ991247
	XM_701685	ENSDARP00000046126		ENSDARP00000070262	
***STICKLEBACK***	ENSGACP00000022696	ENSGACP00000004382	ENSGACP00000002397	ENSGACP00000007119	ENSGACP00000015575
	ENSGACP00000007143	ENSGACP00000016961	ENSGACP00000023187	ENSGACP00000022701	
***MEDAKA***	ENSORLP00000022250	ENSORLP00000011730	ENSORLP00000023740	ENSORLP00000016356	ENSORLP00000020808
	ENSORLP00000016315	ENSORLP00000014962	ENSORLP00000007394	ENSORLP00000022227	
***SALMON***	CK885244	Not identified	CB511922	Not identified	Not identified
***TROUT***	AY706216	AY706218	AY706217 CU069615	Not identified	Not identified

**Figure 4:**
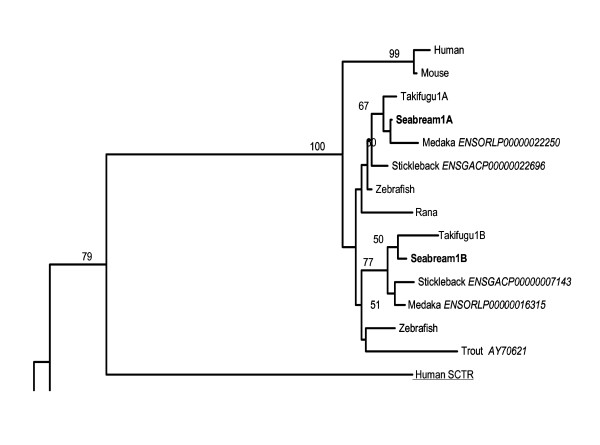
**Phylogenetic analysis of the vertebrate PAC_1 _members**. This figure represents the upper quartile of the consensus tree, for full image and details please refer to Additional file 2. Sea bream PAC_1 _receptors are in bold and the novel teleost PAC_1 _receptors identified are refereed by their Ensembl nomenclature. PAC_1 _accession numbers are: Human, P41586; Mouse, P70205; Rana, Q90Y07; *Takifugu*1A, AJ494861, *Takifugu*1B, AJ490804; zebrafish1A, NM_001013444 and zebrafish1B, XM_701685.

Phylogenetic analysis of sbPAC_1_A and sbPAC_1_B receptors with the vertebrate PAC_1_, VPAC_1_, VPAC_2_, PRP, GHRH and SCT receptors confirmed their identity as duplicated members of the vertebrate PAC_1 _family (Figure 4, Additional file 2). The tree topology indicates that vertebrate PAC_1_, VPAC (1 and 2), PRP and GHRH receptors evolved from a common ancestral gene and that a teleost specific duplication has occurred which is in line with the proposed partial or whole genome duplication event within their lineage [9,12,15,16,32,33].

### Linkage analysis

**Figure 5:**
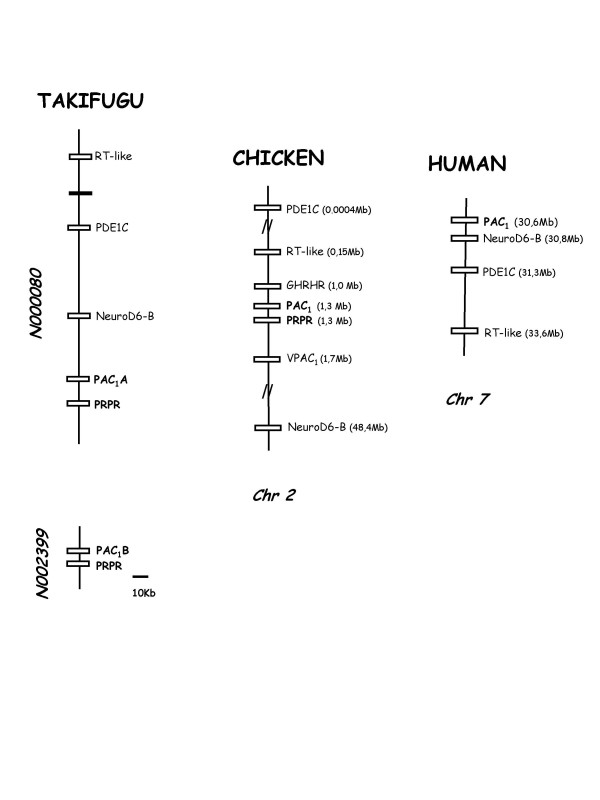
**Short-range linkage analysis of the PAC_1 _receptors**. The gene environment of *Takifugu*, chicken and human PAC_1 _regions is represented. Homologue genes present in all three study organisms are represented by open boxes and black boxes represent flanking genes that had no corresponding homologues. Genes are named according to the HUGO annotation and PAC_1 _genes are in bold. *Takifugu *scaffolds (assembly 4) are represented using the NIX annotation [40]. The gene environment of PAC_1 _in *Xenopus *is not represented since it was very incomplete. The *Takifugu *gene environment is represented and the scale corresponds approximately to 10 Kb and the relative position of linked genes in the chromosomes of the chicken and human is given.

Gene environment comparisons of the PAC_1 _homologue genomic regions revealed it is highly conserved across vertebrates (Figure 5). In human, PAC_1 _is localized on chromosome 7 and in chicken it maps to chromosome 2 and is in close proximity with the chicken GHRH and VPAC_1 _receptors. In teleosts the duplicate genes map to different genome regions and are localised in the *Takifugu *scaffolds N000080 and N00239 and in zebrafish chromosomes 10 and 2. Three genes (RT-like protein, PDE1C and NeuroD6-B genes) were found to be shared between the *Takifugu*, chicken and human genomic regions analysed. The PAC_1 _gene is linked with the PRPR gene in *Takifugu *and chicken suggesting these genes arose by tandem duplication, although it has been lost in the human. In *Xenopus*, in which the genome assembly is incomplete, PAC_1 _maps to the final region of scaffold_333 and NeuroD6-B and PDE1C to scaffold_588 suggesting that these two regions are contiguous in the genome although so far no homologue of PRPR was identified.

### Tissue expression of sbPAC_1 _receptors

**Figure 6:**
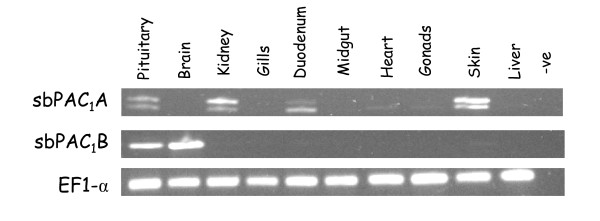
**Tissue expression of the duplicate sbPAC_1_A and sbPAC_1_B receptors**. Expression was carried out by RT-PCR using specific primers to amplify potential receptor transcripts. PCR products of approximately 1200 bp, 1000 bp and 1280 bp were obtained and corresponded to sbPAC_1_A, sbPAC_1_B and sbPAC_1_Ahop1 isoform, respectively.

PAC_1 _tissue distribution was carried out in several sea bream tissues by RT-PCR using specific primers for each receptor (Figure 6). These primers were designed in order to amplify all potential receptor transcripts. The sbPAC_1_A was found to be expressed in the pituitary, kidney, duodenum and skin and weakly in heart and gonads whilst sbPAC_1_B was restricted to brain and pituitary. A larger sbPAC_1_A PCR product was also amplified which had an overlapping tissue distribution with sbPAC_1_A with the exception of heart and gonads. Sequence analysis indicates that the larger sbPAC_1_A PCR product corresponds to the homologue of the mammalian PAC_1_hop1 isoform and contains an insertion of 28 amino acids within IL3 region. No other PAC_1_A isoforms were amplified and no alternative splice forms for sbPAC_1_B were detected.

### Functional studies of duplicate sbPAC_1_

The sbPAC_1_A and sbPAC_1_B were successfully expressed in Cos7 and Hek293 cell lines as confirmed by immunofluorescence and Western blot analysis. Cos7 cell extracts subject to Western blot contained a specific immunoreactive fusion protein of approximately 52 kDa in transfected cells, which corresponds to the estimated molecular weight *in silico *of the fusion proteins, (T7PAC_1_A is 52.75 kDa and T7PAC_1_B is 52.32 kDa). Cos7 and Hek293 cell lines expressing the recombinant vector were activated by Forskolin and gave maximal cAMP production. Negative control experiments in which Cos7 and Hek293 cells were transfected with pcDNA3 without insert and incubated with the maximum concentration used of test peptide (10^-6 ^M), revealed that Hek293 cells are responsive to PACAP and VIP peptides. This suggests the existence of endogenous receptors in Hek293 which is confirmed by available proteome data [34]. For this reason only the results obtained with transfected Cos7 cells are presented.

**Figure 7:**
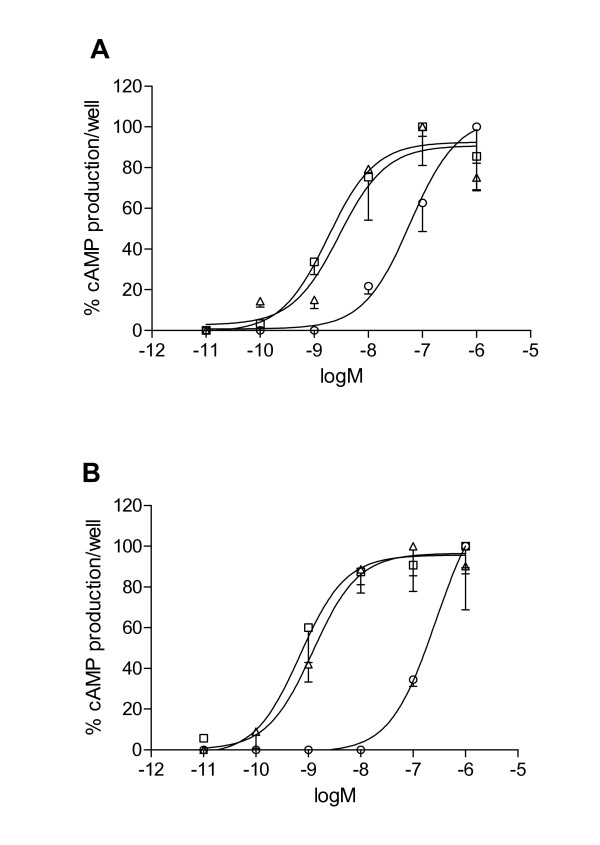
**Production of maximal cAMP by the recombinant Cos7 cell expressing sbPAC_1_A (A) and sbPAC_1_B (B)**. Transfected cells expressing the receptors were incubated with different concentrations of PACAP_27 _(□) PACAP_38 _(△) and VIP (○). Data was normalized and receptor potency profiles is given as percentage of intracellular cAMP produced per well and error bars indicate the ± SEM of a minimum of three independent experiments performed in duplicate. Only the lower error bars are represented.

The cAMP potency profile of Cos7 transfected cells expressing the recombinant sbPAC_1 _receptors in the presence of different concentrations of VIP and PACAP (PACAP_38 _and PACAP_27_) peptides is depicted in Figure 7. All mammalian peptides were able to stimulate cAMP production in a dose dependent manner. The peptide activation profile for the sbPAC_1_A gene was as follows PACAP_27 _≈ PACAP_38 _> VIP as assessed by the LOG_EC50 _values for cAMP production for each peptide (-8.74 ± 0.29 M, -8.54 ± 0.18 M and -7.23 ± 0.20 M, respectively). The stimulation of cAMP production by VIP was significantly lower (P < 0.05) than for PACAP peptides. The peptide activation profile of sbPAC_1_B was also tested and found to be similar to sbPAC_1_A and is as follows PACAP_27 _≈ PACAP_38 _> VIP and the LOG_EC50 _values for maximal cAMP production for each peptide are -9.15 ± 0.21 M, -8.92 ± 0.24 M and -6.57 ± 0.14 M, respectively. The stimulation of cAMP by VIP was significantly lower than PACAP_27 _and PACAP_38 _(P < 0.001). Human SCT causes a negligible increase above the basal concentration of cAMP production in non-stimulated Cos7 cells transformed with either sbPAC_1_A or sbPAC_1_B (data not shown). Comparison of the potency profile of each peptide (PACAP_27_, PACAP_38 _and VIP) for the duplicate sea bream receptors revealed that PACAP stimulates both receptors equally. In contrast, VIP is significantly more potent for sbPAC_1_A (-7.23 ± 0.20 M) compared to sbPAC_1_B (-6.57 ± 0.14 M, P < 0.05).

## Discussion

In the present study, duplicate sea bream PAC_1 _genes (sbPAC_1_A and sbPAC_1_B) have been isolated and functionally characterised. Structural motif identification and expression assays reveal they are functional family 2 GPCR members. The sbPAC_1_B is predominantly found in brain and pituitary while sbPAC_1_A has a widespread distribution but is absent from brain. The overall tissue distribution of the sbPAC_1 _receptors is similar to the single mammalian PAC_1 _receptor and suggests they may have similar functions in the endocrine, nervous, gastrointestinal and reproductive systems [17,18]. In common with the mammalian PAC_1 _receptor both sbPAC_1 _receptors are highly stimulated by PACAP but poorly by VIP and SCT. The tissue distribution of PAC_1 _receptors in sea bream is different from that in *Takifugu *in which PAC_1_B has a widespread tissue distribution and PAC_1_A distribution is restricted to brain, gill and gonads [15]. Therefore, despite the high sequence conservation between teleost PAC_1_A or PAC_1_B genes (91% and 86%, respectively), the paralogue genes seems to have evolved in a species specific manner in relation to tissue distribution and possibly function.

Duplicate PAC_1 _genes have been identified in other teleosts and also for other family 2 GPCR members [14,15] as expected in light of the proposed partial or full genome duplication event suggested to have occurred within the teleost lineage [32,33,35-37].

Analysis of teleost genomes indicates that duplicate PAC_1 _genes are linked with the recently reclassified GHRH-like receptor which based upon ligand binding characteristics has been reassigned as a teleost PRP receptor (PRPR) [38,39]. In *Takifugu*, PAC_1_A and PRPR genes are localised on scaffold N000080 and PAC_1_B/PRPR in scaffold N002399 and in the zebrafish genome on chromosome 10 and 2, respectively [40,41]. In terrestrial vertebrates, with the exception of mammals, co-localization of both receptors is also observed suggesting that PAC_1 _and PRPR genes arose by tandem gene duplication prior to the teleost divergence and that PRPR gene was subsequently lost in the mammalian lineage (Figure 6).

Stimulation of cAMP production and not peptide affinity or activation of alternative signalling pathways has been investigated and revealed that sbPAC_1 _receptors are highly stimulated and have identical potency profiles for the mammalian PACAP_27 _and PACAP_38 _peptides compared to VIP. The latter peptide was found to be more potent for sbPAC_1_A in comparison to sbPAC_1_B. Although, the identification in teleosts of duplicate genes for the ligands and the existence of two potentially active PACAP peptides raises further issues in relation to PAC_1 _receptor activation and function. Two copies of PACAP are present in *Takifugu *(DQ659331 and DQ659332), *Tetraodon *(Q4RN19 and Q4RH43) and zebrafish (NW_652622 and NW_634478), but functional studies with the duplicate receptors and their ligands are scarce. The ligand binding characteristics of the duplicated zebrafish zfPACAP_27 _peptides for the zebrafish PAC_1 _receptor, the sequence homologue of sbPAC_1_A, was tested and both peptides strongly stimulate in a similar way the cAMP and phospholipase pathways [28,42] suggesting conservation of function. A further measure of complexity is also introduced by the identification of duplicate VIP genes in teleost genomes [38,43] and it will be of importance in future to establish the affinity of the duplicate peptides for the duplicate receptors, as well as compare their tissue distribution in teleosts.

Moreover, a microsatellite is identified for the first time in the 5'UTR of PAC_1 _receptors. Genotyping analysis of the microsatellite reveals it is polymorphic in sea bream and raises intriguing questions about its potential influence on gene expression. Analysis of homologue regions in other teleosts [41] also reveals the presence of a microsatellite in the 5'UTR. For example, in the stickleback PAC_1_A and PAC_1_B respectively, two perfect microsatellite repeats (TG)_21 _and (CA)_34 _and an imperfect (CA)_21 _dinucleotide repeat are present. The PAC_1_A gene in medaka contains a (TG)_8 _repeat, although no microsatellite is present in the paralogue gene (Additional file 3). So far no microsatellites have been described or identified in the mammalian, chicken, frog and goldfish homologue receptors [28,44-46]. The importance of PACAP in growth and development [17,18] and the presence of a microsatellite in its receptor suggests it may be a useful tool for genomic and functional analysis. A previous study of early growth variation in the Artic charr (*Salvelinus alpinus*, [47]) revealed a strong marker-trait association with a single nucleotide polymorphism (G/A) of the 18^th ^base pair of the intronic region between the exons that code for PACAP-related peptide and PACAP in the PRP/PACAP gene precursor.

The human SCT peptide failed to significantly stimulate cAMP production by the sbPAC_1 _receptor which may indicate either a failure to activate the cAMP signalling pathway or a failure to bind the receptor. The preceding results are at odds with the physiological role attributed to secretin in fish, pancreatic stimulation via cholecystokinin and oxyntomodulin [48]. However, SCT has only been identified by immunohistochemistry in the gastrointestinal tract of the flower fish (*Pseudophoxinus antalyae*) [49]. The failure to identify in teleosts a gene for the SCT receptor or its ligand suggests they probably evolved subsequent to teleost divergence or were lost during the teleost radiation and the significant sequence differences between SCT and VIP or PACAP probably explains the absence of activity of SCT in the present study [16].

Gene duplicates and generation of alternative splice isoforms are major contributors to functional diversity of the vertebrate proteome. In teleosts, the existence of gene duplicates is proposed to reduce the incidence of gene isoforms since they are assumed to generate functional redundancy of single-copy gene splice isoform [50], and this may explain reduced number of PAC receptor splice isoforms detected in the sea bream. In sea bream, a PAC_1_Ahop receptor isoform was identified with an overlapping tissue distribution with the shorter PAC_1_A receptor transcript, but it was different from the tissue distribution reported for alternative splice isoforms in zebrafish and goldfish [26,28]. Recently in zebrafish, two novel IL3 splice isoforms were characterised, a hop2 isoform (insertion of 87 bp) present in ovaries and a novel skip isoform (resulting in a truncated protein) in the gills [28]. Characterisation of a recently isolated PAC_1 _and hop1 receptor isoform in the goldfish reveals that they have similar activation profiles raising question about their functional role [26]. The mounting evidence for the presence of hop1 receptor isoforms in all vertebrates suggests that a homologue transcript was probably also expressed by the PAC_1 _receptor gene in ancestral metazoa [50]. In order to understand the functional relevance of duplicate receptors and isoforms it will be important to establish if they are co-localised *in vivo *in order to establish possible interactions.

Expression studies of the sbPAC_1 _genes demonstrated they are functional family 2 GPCRs and PACAP and VIP peptides were able to stimulate cAMP production in a dose dependent manner. Such assays also indicate that both sea bream receptors are specific PACAP receptors and have different activation profiles for VIP although, as only cAMP production was measured, it remains to be established if a similar activation profile occurs for the alternative IP3 signalling pathway. One hypothesis for the apparent functional divergence and differential expression of sea bream paralogue receptors may be related to functional specialisation related to the existence in teleosts of duplicate ligands which might also explain the significant amino acid changes (48% sequence identity) observed in the N-terminal region. In mammals, important amino acid motifs involved in receptor binding have been identified and mutation studies reveal the importance of the N-terminus [20]. Amino acid motifs involved in ligand binding are conserved between tetrapod and teleost homologue receptors and include the motifs W-D, G-W-S and the following amino acids W, P and P (Figure 3). However, the amino acid motifs that account for potential ligand selectivity of the teleost duplicated receptors are still unknown, future receptor mutation studies should help to clarify this issue.

An intriguing aspect about the sbPAC_1 _genes is their divergent tissue distribution and this may be the basis of their specific physiological functions and persistence in the genome. Understanding the biological function of receptors is complex as it is not only the availability and concentration of receptors but also of ligands and accessory factors at a given site which will determine receptor preference/activity and ultimately biological function. Physiological studies of the ligands, PACAP and VIP, in teleosts are not very numerous and certainly do not encompass all the actions assigned in mammals making it difficult currently to assign possible biological roles to the duplicate receptors. However, one major function attributed to PACAP is its role in GH-release [46,51,52]. In common with mammals, PACAP_38 _is the predominant isoform in teleost brain and this peptide is found to have a more potent stimulatory effect on fish GH secretion by pituitary cells when compared to GHRH and GnRH (potent mammalian GH releasing factors). In contrast, VIP has little or no effect on GH release [46,51,52] and this has been taken to suggest that teleost GH-release is mediated via PAC_1 _pituitary receptors and the sea bream PAC_1_B may play a central role in this process. Relatively few studies have been carried out to characterise the biological activity of fish VIP and as in mammals it is proposed to have an important role in the gastrointestinal system [51,53,54]. In cod, *Gadus morhua*, VIP stimulates gastric and pancreatic secretion [55,56] and in tilapia it is involved in ion and water absorption by the intestine [57]. In sea bream, tissue distribution of PACAP and VIP transcripts indicate that PACAP is mainly restricted to nervous tissue whilst VIP is abundant in the gastrointestinal system but is also present in a wide range of other tissue (unpublished data). The widespread distribution in non nervous tissue of sbPAC_1_A indicates that this receptor may have a broader physiological role. Unquestionably much more work is required to elucidate the physiological relevance of duplicate sbPAC_1 _and alternative approaches such as ligand mutational studies; gene knock-down strategies and physiological experiments will be needed.

## Conclusion

Duplicate PAC_1 _receptors genes are present in the majority of teleost genomes, although the reason for their persistence is not yet clearly established. The present study with duplicate sbPAC_1 _receptors suggests that their maintenance may be due to a process of neofuncionalisation as a consequence of the accumulation of mutations in the ligand binding domain of the receptors after duplication. Such a proposal is supported by the poor sequence conservation (48%) in the N-terminal ligand binding domain of the receptor. The divergent tissue distribution of the receptors, with one form predominantly found in nervous tissue and the other with a more widespread distribution is highly suggestive of functional divergence. The isolation and characterisation of ligands for the receptors in teleosts will be an important step in establishing receptor function, as will improved characterisation of the tissue distribution of both receptors and ligands and the factors regulating their expression.

## Methods

### Sea bream cDNA library screening

A homologous PAC_1 _receptor probe (620 bp) was obtained by RT-PCR using sea bream brain cDNA and degenerate primers flanking transmembrane regions 2 and 7 (Table 2). PCR reactions were performed in a 25 μl reaction volume with 1 × PCR buffer (Biocat, Italy), 1,5 mM MgCl_2 _(Biocat), 0,2 mM dNTPs (GE healthcare, UK), 1 mM of each primer, EuroTaq DNA Polymerase 5 U/μl (Biocat) and DNase Free water (Sigma-Aldrich). The PCR product was used to screen sea bream pituitary and kidney cDNA libraries constructed using the λ-Zap vector II kit (Stratagene, USA) [58]. The phage libraries were titred to obtain approximately 500,000 pfu per plate and blotting procedures were performed in duplicate using Nylon membranes (Hybond-N, GE healthcare, UK). Probes were radioactively labelled using α-P^32^dCTP (GE healthcare, UK) and the Rediprime kit (GE healthcare, UK) following manufacturer's instructions. Membrane filters were hybridised overnight at 58°C with Church-Gilbert hybridisation solution (1 mM EDTA pH8; 0.5 M NaPO_4 _pH7.2, 7% (w/v) SDS) and washed under high stringency conditions at 58°C and exposed to X-OMAT film (Kodak, USA) at -80°C for 24 hours. Positive clones were selected based on the intensity of signals and their presence on the duplicate plate. Single clones were excised into phagemid vectors using the manufacturer's protocol and DNA was extracted and sequenced to confirm its identity.

**Table 2:** Primer sequences used for PCR amplification reactions.

	**PRIMERS**	
***Probe synthesis***		
PAC_1_	*TM2fwd *ct(g/t)(a/t)tt(g/t)tgtccttcatcctga	*TM7rev *ac(a/c)acaaarccctg(a/g)aagga
***Expression studies***		
SbPAC_1_A	*713T31F *cgagcgatgaccttgagttag	*713T3R4 *gggaggctgatgttggcgtt
SbPAC_1_B	*1CF5 *catacggacacatactatgca	*1CR4 *agtggccggggtctcggc
EGF	EGFαfwd cgctgtgacaacctgctg	*EGFαrev *agttccaataccgccgat
***Cloning***		
SbPAC_1_A	*pcDNA3CD33T7NFwd *ccgagatctagagtccgagcactgg	*pcDNA3CD33T7NRev *ggcgaattctcaggtggggaggctgat
SbPAC_1_B	*pcDNA3CD33T7NFwd *ccgagatctacaacaggtctcttcaaac	*pcDNA3CD33T7NRev *ggcgaattctcaagtggccggggt
***Microsatellite analysis***		
SbPAC1A	*SpauPK713F *ggagtgtgttgccgctga	*SpauPK713R *gtatccaaaaggctccacga
SbPAC1B	*SpauPP1CF *acccgttcatttcgcaca	*SpauPP1CR *ttcgccctccacacaaga

### RT-PCR analysis of receptor tissue distribution

Tissue distribution and analysis of PAC_1 _splice variants was carried out by RT-PCR. Total RNA was extracted from sea bream pituitary, brain, kidney, gills, gut, heart, gonads, liver and skin using TRI reagent (Sigma-Aldrich, Spain). Mature sea bream of 11–13 months old weighing approximately 350–500 g were sacrificed by decapitation and their tissues collected and immediately frozen at -80°C. cDNA was synthesised using 1 μg of sea bream total RNA and a reverse transcription system kit (Promega, Spain) and hexameric oligonucleotides following the manufacture's instructions. The quality of cDNA obtained was verified by PCR with sea bream EF1-α (a housekeeping gene) using the following thermocycle; 94°C for 2 minutes; 25 cycles (94°C for 1 minute, 58°C for 1 minute and 72°C for 1 minute) and a final extension step at 72°C for 5 minutes. Specific primers for each sbPAC_1 _gene were designed (Table 2) and PCR was performed as previously described using the following cycle: 94°C for 2 minutes; 34 cycles (94°C for 1 minute, 62°C for 1 minute and 72°C for 1 minute) and a final step at 72°C for 5 minutes. The products obtained were sequenced to confirm their identity.

### Genotype analysis

The variability of a microsatellite identified in the 5' UTR of sbPAC_1_A and sbPAC_1_B was assessed using specific primers (Table 2) and sea bream genomic DNA from fish of diverse geographic origins (Morocco, Portugal, France and Adriatic sea) and from a genomic panel composed of parents and 50 first generation progeny. PCR reactions were performed using fluorescently labelled forward primers (0.3 μM; 6-FAM and TET; Metabion International AG) and unlabelled reverse primers (0.3 μM) using the following thermocycle: 95°C for 2 minutes; 35 cycles (95°C for 30 seconds, 57°C for 30 seconds and 72°C for 30 seconds) and a final step at 72°C for 5 minutes. PCR products were separated using high resolution 6% Long Ranger acrylamide gels (Cambrex, USA) on a automated ABI 377 sequencer (Applied Biosystems, USA) and data was analysed with the GenScan software (Applied Biosystems, USA).

### Database searches, sequence alignments and phylogenetic analysis

The conserved amino acid sequence of sbPAC_1 _transmembrane (TM) domains was used to search for homologous receptors in teleost genomes. Briefly, the amino acid sequences of the seven TM domains were extracted, concatenated and used in BLAST sequence similarity searches [59] against the *Tetraodon nigroviridis *[41], medaka (*Oryzias latipes*), stickleback(*Gasterosteus aculeatus*), zebrafish (*Danio renio*) [41] and atlantic salmon (*Salmon salar*) [60] genome databases and NCBI EST database [61]. The amino acid sequence of the TM domains of a total of 52 receptors were concatenated and a multiple sequence alignment was produced using the ClustalX vs1.83 (Blosum matrix and Gap opening penalty 10 and Gap extension 0.2) [62] and percentage of identity/similarity calculated using Genedoc [63]. The alignment produced (length 170, with 162 informative sites) did not require the insertion of gaps and was used to construct phylogenetic trees using both maximum parsimony and neighbour joining methods [64] with 1000 bootstrap replicates, complete gap deletion and Poisson correction in MEGA 3.1 phylogenetic programme [65].

### Short-range linkage analysis

The gene environment of the *Takifugu*, *Xenopus*, chicken and human PAC_1 _homologue regions were compared using a sequence similarity approach. The human and chicken gene environments were accessed using the NCBI Mapview interfaces [61] and *Xenopus *using the Ensembl database [41]. The gene environment of the *Takifugu *scaffolds (release17/05) was accessed using NIX annotation [40] and the neighbouring genes were used to search for orthologues in human, chicken and *Xenopus *genomes using the tblastn algorithm [61].

### Construction of the recombinant expression vector

Specific primers for each sbPAC_1 _gene were designed to amplify the mature receptor sequence (Table 2) and were cloned into pcDNA3 vector (Invitrogen, UK) containing a signal peptide of CD33 and a T7-epitope tag (CD33-T7-pcDNA3, [66]). In order to facilitate cloning, restriction digestion sites for *BglII *and *EcoRI *enzymes were incorporated in the forward and reverse primers respectively. Template amplification was carried out using the thermocyle; 95°C for 2 minutes; 35 cycles of (95°C for 1 minute, 68°C for 1 minute and 70°C 2 minutes); 72°C for 10 minutes with a proof-reading *Pfu *DNA polymerase (Promega, Spain) in a 25 μl reaction volume containing 1 × PCR buffer (Promega, Spain), 1,5 mM MgCl_2 _(Promega, Spain), 0,2 mM dNTPs (Amersham), 1 mM of each primer and 1,5 U *Pfu *DNA polymerase (3 U/μl)) and DNase Free water (Sigma-Aldrich, Spain). The amplified PCR products were cloned into pGEMT-easy vector (Promega, Spain), sequenced and subcloned into CD33-T7-pcDNA3 vector in frame with the T7-epitope tag. The recombinant constructs produced (CD33-T7-pcDNA3+sbPAC_1_A and CD33-T7-pcDNA3+sbPAC_1_B) were sequenced and used to transfect mammalian Cos7 and Hek293 cells lines. The CD86 (NM_175862) membrane protein of the immunoglobulin superfamily cloned in CD33-T7-pcDNA3 expression vector was used as positive control for cell transfection (de Vet et al., 2001).

### Mammalian cell transfections

Mammalian Cos7 and Hek293 cell lines were transfected using the Effectene transfection kit (Qiagen, Germany) and the success of transfection was assessed by western blot and immunofluorescence assays using antisera raised against the T7-epitope tag protein [66]. One day prior to transfection, approximately 60,000 to 70,000 Cos7 and Hek293 cells were seeded and transfections carried out using approximately 0.4 μg of recombinant construct (pcDNA3+sbPAC_1_A or B) and cells were grown for 2 days at 37°C. The viability of transfected cells was determined by dye exclusion using Trypan blue (0,4% solution, Sigma) and the success of transfection calculated by fluorescent confocal microscopy using DAPI (4',6-Diamidino-2-phenylindole) and FITC (Fluorescein) staining for Cos7 cells and the FACS (Fluorescence Activated Cell Sorting) method for Hek293 cells. The percentage of cell transfection was estimated to be approximately 30% in both cell lines.

### Immunofluorescence and western blot assays

An anti-T7 epitope monoclonal antibody (Novagen, UK) was used in immunofluorescence and western blots assays to assess the success of transfection and production of recombinant fusion protein (T7+sbPAC_1_) as described in de Vet et al., 2001 [66]. Briefly, for the immunofluorescence assay, cells were grown in 6 well plates (Greiner, Germany) on sterile cover slips. Approximately 120,000 cells were used per well and cell transfections were carried out using 0.4 μg of DNA as described above. Immunofluorescence localization studies were performed [67] and DAPI and FITC staining examined using a microscope linked to a confocal imaging system (Bio-Rad, UK). Western blots were carried out by lysing transfected cells (30 μl) in 1× Laemmli SDS-PAGE loading buffer [68] and proteins were fractionated on a 10% SDS-PAGE gel with a constant current of 35 mA. The fractionated proteins were transferred to a nitrocellulose membrane (GE healthcare, UK) and blocked (2% milk powder in 1 × PBS/0.1%Tween) for 1 hour at room temperature. Incubations were carried out with anti-T7 tag monoclonal antibody (Novagen, UK) and a secondary antibody coupled to Horse-Radish peroxidase as described in de Vet., 2001 [66]. Immunoreactive proteins were detected using the ECL system (PerkinElmer Life Sciences, UK). The size of the immunoreactive protein was comparable to the estimated sizes of the recombinant receptor protein determined *in silico *using the Swiss Prot database interface [69].

### Ligand-binding studies

Ligand binding studies were performed two days after transfections, in three independent experiments. Transfected cells were incubated in duplicate with different concentrations (10^-6 ^to 10^-11^M) of the human VIP, PACAP_38 _and secretin (SCT) peptides and ovine PACAP_27 _peptide for 30 minutes (Sigma-Aldrich, Spain). Homologous sea bream peptides are unavailable but their predicted amino acid sequences are 82%, 100%and 92% identical to human VIP, PACAP_27 _and PACAP_38 _respectively and important amino acids at N-terminus potential involved in receptor binding are totally conserved (unpublished data). Prior to ligand-binding assays, Cos7 and Hek293 cells were washed 3 times with DMEM medium without fetal calf serum and incubated in a CO_2 _incubator with 1 mM of IBMX (3-Isobutyl-1-Methylxantine) for 30 minutes. Ligand-binding assays were performed by incubating the cells in fresh medium containing the peptides in the presence of 1 mM IBMX for 30 minutes at 37°C in the CO_2 _incubator. After incubation, cells were lysed and stored at -80°C until required. Mammalian Cos7 and Hek293 cells transfected with wild type pcDNA3 were used as negative controls which were incubated with the highest tested peptide concentration (10-^6^mM). Prior to assays the responsiveness of transfected Cos7 and Hek293 cells was tested by stimulating cAMP production using Forskolin (10 mM, 1 mM and 0.1 mM; Sigma-Aldrich).

### Radioimmunoassay (RIA) and statistical analyses

The quantification of cAMP produced was determined by radioimmunoassay using the TRK432 kit (GE Healthcare, UK) following the manufacturer's instructions. Cos7 and Hek293 cells were lysed by sonication, centrifuged and the supernatant heat denatured for 10 minutes at 100°C. The concentration of cAMP produced (pmol/well) was determined in duplicate for each sample and calculations performed based on a linear regression curve constructed using standard concentrations of labelled (^3^H) cAMP. The cAMP data was normalized as a percentage of stimulation above basal levels and plotted as a percentage of cAMP production per well (%cAMP/well). The results are presented as the mean ± SEM of three independent experiments in duplicate and analysis was performed using the SigmaPlot9.01 programme. Data was analyzed by comparing the potency profile of each sbPAC_1 _receptor in the presence of different concentrations of test peptides and by comparing the potency profile of both receptors in the presence of identical peptide concentrations. The presence of significant differences in cAMP production was assessed with the SigmaStat3.11 programme using two way Anova and the Holm-Sidak method for pairwise multiple comparisons (P < 0.05 and P < 0.001 were considered statistically significant).

## Authors' contributions

The majority of the work described was carried by JCRC and the cell expression studies were carried out in collaboration with ECJMV and GE. BL carried out the genotype analysis. MSC and DMP planned the study, discussed the results and critically revised the manuscript for important intellectual content and data analysis. All authors read and approved the final manuscript.

## Supplementary Material

Additional file 1 **Genotyping analysis of the sbPAC_1 _microsatellites in a geographic (A) and family (B) panels**. Score alleles for sbPAC_1_A are represented from A-D and for sbPAC_1_B from A'-F'. In A, individuals were sampled in four distinct geographic regions. Three in the Atlantic Ocean (Atl) in the Moroccan (Mr), Portuguese (Pt) and French (Fr) coast and in the Adriatic Sea (Adr) in Italian coast. In B, M represent males and F females and 50 progeny individuals were analysed and the percentage of allele scores is indicated. Click here for file

Additional file 2 **Phylogenetic analysis of PAC_1_/VPAC/PRPR/GHRHR family members**. Sea bream PAC_1 _genes are indicated in bold and human secretin receptor (SCTR, Q8IV17) is underlined. The receptors identified in *Tetraodon *(GSTENP00011829001, GSTENP00034495001 and GSTENP00011830001), Stickleback (ENSGACP00000002397), Medaka (ENSORLP00000007394), zebrafish (ENSDARP00000054330 and ENSDARP00000070262), salmon (CK885244 and CB511922) and trout (CU069615) were not used in the analysis since they were found to have incomplete TM domains. Accession numbers for *Takifugu *and zebrafish receptors were obtained from Table 1 and the novel genes identified in ENSEMBL are indicated. Receptors accession numbers used are: Human PAC_1_, P41586; Mouse PAC_1_, P70205; Rana PAC_1_, Q90Y07; Human VPAC_1_, P32241; Rat VPAC_1_, P30083; Rana VPAC_1_, Q9YHC6; Human VPAC_2_, P41587; Rat VPAC_2_, P35000; Chicken PRPR, XM_425958; Goldfish PRPR, O73768; Human GHRHR, Q02643; Mouse GHRHR, Q8AXV2. Bootstrap values under 50 were removed. Click here for file

Additional file 3 **Sequence of the putative 5'UTR regions of the Stickleback and Medaka duplicate PAC_1_s genes**. Sequences were obtained from ENSEMBL database [41] and 3000 bases localised upstream the initial methionine are represented. Microsatellite repeats identified are underlined and in italics and in bold upper cases the sequence of the first predicted exon is represented. Click here for file
