# Supplementary material for: Persistence of duplicated PAC1 receptors in the teleost, Sparus auratus
Source: BMC Evol Biol. 2007 Nov 12;7:221. doi: 10.1186/1471-2148-7-221 (PMC2245808; doi:10.1186/1471-2148-7-221)
Supplement: Additional file 1 — Genotyping analysis of the sbPAC1 microsatellites in a geographic (A) and family (B) panels. Score alleles for sbPAC1A are represented from A-D and for sbPAC1B from A'-F'. In A, individuals were sampled in four distinct geographic regions. Three in the Atlantic Ocean (Atl) in the Moroccan (Mr), Portuguese (Pt) and French (Fr) coast and in the Adriatic Sea (Adr) in Italian coast. In B, M represent males and F females and 50 progeny individuals were analysed and the percentage of allele scores is indicated. [file 1471-2148-7-221-S1.pdf]

Additional file 1: Genotype analysis of the sbPAC1 receptors in a geographic (A) and family (B) panels

A) Geographic panel

Individuals Origin	$sbPAC_1A$	$sbPAC_1B$
Atl Mr	A/B	B'/D'
Atl Pt	B/C	A'/H'
Atl Fr	A/A	E'/F'
Adr It	A/A	E'/G'

B) Family panel

	Loci Alleles					
**Linkage Panel**	**$sbPAC_1A$**		**$sbPAC_1B$**			
**Parents**	**M1** B/B	**F1** A/D	**M1** B'/E'	**F1** C'/E'		
**Progenies**	**A/B**	**B/D**	**B'/C'**	**B'/E'**	**C'/E'**	**E'/E'**
n=50 individuals	48%	52%	31%	35%	17%	17%
