# Supplementary figures and images for: Persistence of duplicated PAC1 receptors in the teleost, Sparus auratus
Source: BMC Evol Biol. 2007 Nov 12;7:221. doi: 10.1186/1471-2148-7-221 (PMC2245808; doi:10.1186/1471-2148-7-221)

**Additional file 2: Phylogenetic analysis of PAC1/VPAC/PRPR/GHRHR family members.**

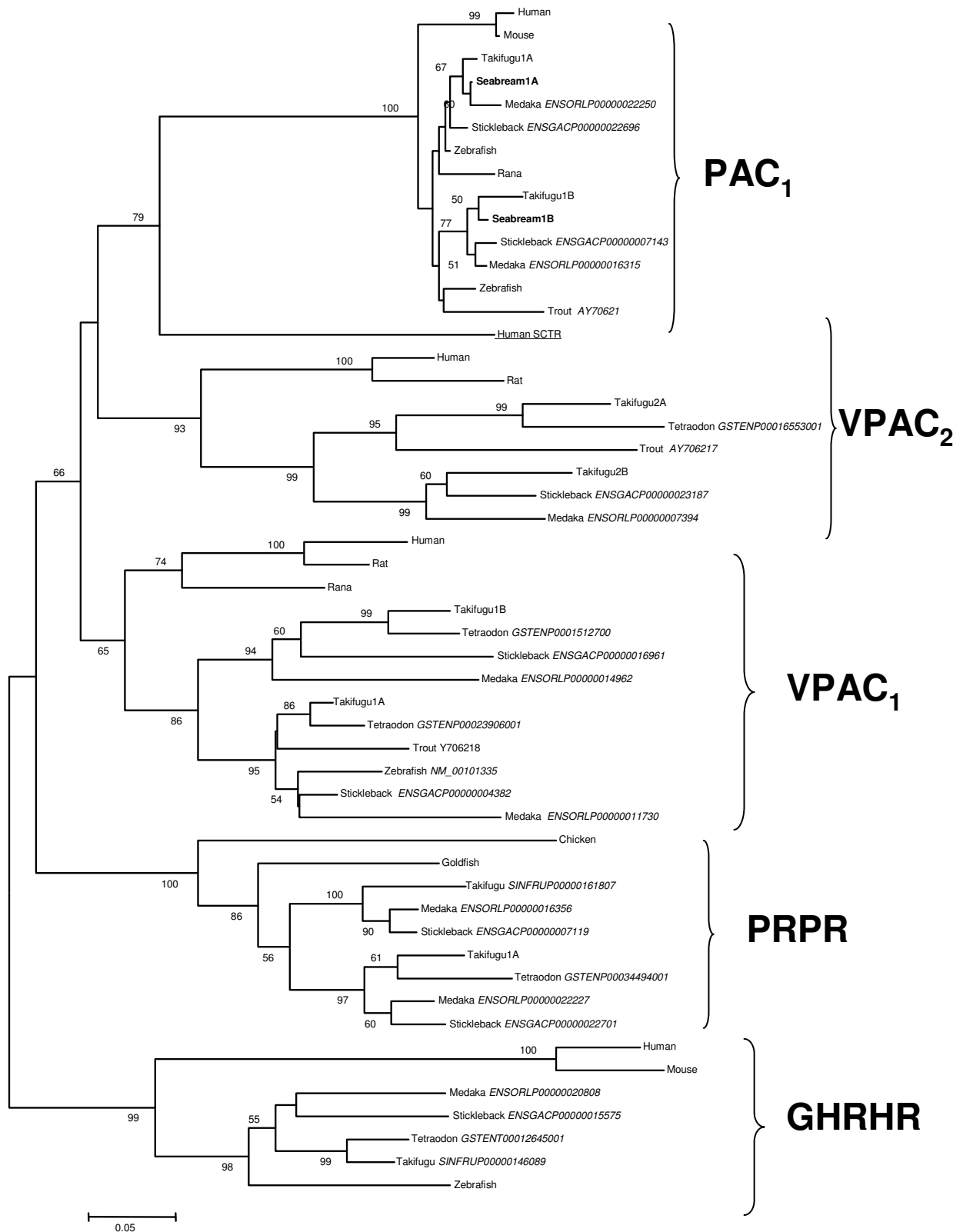

Supplement: Additional file 2 — Phylogenetic analysis of PAC1/VPAC/PRPR/GHRHR family members. Sea bream PAC1 genes are indicated in bold and human secretin receptor (SCTR, Q8IV17) is underlined. The receptors identified in Tetraodon (GSTENP00011829001, GSTENP00034495001 and GSTENP00011830001), Stickleback (ENSGACP00000002397), Medaka (ENSORLP00000007394), zebrafish (ENSDARP00000054330 and ENSDARP00000070262), salmon (CK885244 and CB511922) and trout (CU069615) were not used in the analysis since they were found to have incomplete TM domains. Accession numbers for Takifugu and zebrafish receptors were obtained from Table 1 and the novel genes identified in ENSEMBL are indicated. Receptors accession numbers used are: Human PAC1, P41586; Mouse PAC1, P70205; Rana PAC1, Q90Y07; Human VPAC1, P32241; Rat VPAC1, P30083; Rana VPAC1, Q9YHC6; Human VPAC2, P41587; Rat VPAC2, P35000; Chicken PRPR, XM_425958; Goldfish PRPR, O73768; Human GHRHR, Q02643; Mouse GHRHR, Q8AXV2. Bootstrap values under 50 were removed. [file 1471-2148-7-221-S2.pdf]
