# Supplementary material for: Persistence of duplicated PAC1 receptors in the teleost, Sparus auratus
Source: BMC Evol Biol. 2007 Nov 12;7:221. doi: 10.1186/1471-2148-7-221 (PMC2245808; doi:10.1186/1471-2148-7-221)
Supplement: Additional file 3 — Sequence of the putative 5'UTR regions of the Stickleback and Medaka duplicate PAC1s genes. Sequences were obtained from ENSEMBL database [41] and 3000 bases localised upstream the initial methionine are represented. Microsatellite repeats identified are underlined and in italics and in bold upper cases the sequence of the first predicted exon is represented. [file 1471-2148-7-221-S3.pdf]

Additional file 3: Sequences of the putative 5´UTR regions of the stickleback and medaka duplicate PAC_1 genes

STICKLEBACK PAC_1s

ENSGACT00000022739

tctctctcagctcaacatcccggggtcgagacttaaaaaagcatccaccgcatttccagactaaacggttgtctcact
ttgttgtctctgtgtctgacgaggtgcgaatcccaaaggaacctcccactcctcctcctcccactcctcctcctc
ctcctccacgccgtcgtggtgccgcagcagccagcgcggtgagtgcgtcctgcagcagcagcagcagcaacaacaaca
gcagcaaaaacggcacctgtcgtttctcttgacgagacgataacgattttaggagtgcaatctattattgcacaagta
aatcaaggtattctcgtcggggattctgcctgaatgcagttgtgcagatatttgcaccgctgaaaattacgcatcggt
aagttcttctgacttgttttgcacgcccggcggctcgcgctttcacgcacatctttgcggcggatcaattaagcccga
tgctttgcgttaaagtagacgtgcatgtccgtattcaagctggagactcgtttttgcgtgtgaaagtgttcgcggcag
acggtgctgaggtttttccatgtgcatagcgaaggtcatccacacgcaaagaggtattacgcgttcacgaatgcggtg
atgatgaaggcgggatgcggggcaatgcgagcccactgattccgcaccgccaagggggggggagacatttatcactg
attgtatttaagactgttgcatttttctccaacatactgtttgtcgttttgcaagctgtgctctaaataataacacaa
tagcctgtaataactgtggatgtaaatggtgactgtaatgcagccgttgtggtcattagcaaacagtaaatgaacatg
ttaacaaagcagccaatacattttggattggcactgtgcacgctgacagacatgcaatccctctgtaaaggcattaac
acacttgcatctgggttaattgcaagcgtccttctgtattacagggatcggaatgaagtgaaaatgaatcattgatgc
accaccatgttcaaaggaatgcaacctgcatcaggaggaagctgatgtggggaaacatcataaaaaataaccctttac
gatagagacaaatattaaggagtgctttccccttctcttgttgttcctgtgtatctgcaaagtacacacagcttgtt*c*
*acacacacacacacacacacacacacacacacacacacacacacacacacacacacacacacacac*tggatgtggt
tctcgatcttctcatcaagggttgaagtctgtaattattcccagaacgtagtgtttggtcacgttcagccaatgtgtg
tcttccattaccgtatgaaataaacaatactgtggtttctgtagacccacacatctccacttcctgtctttaactggt
catgattcgtttttccgcttaaatttccatccctgtgaagcttcatcgacgtgcaccccgtgctgagtaaagcaaag
actctttttctcccttgctcttagaatggttttgacggtggccccttcggtcgtttcctgcaagaataccgacgtgtt
cttttacttcattcggaatctccagacgcacgccccttttttatgggttagataaaaacaaatgcagctgaatgacgcc
acgtctggcaagttcctctctcctctccttctgtttttcagaattaaactccatctggacagtttcatttcagttgcc
ccccaaaatctcaaggtctttcctctgagcacggtcggccctaagaagcatccgtcggaagctttagagctaattaga
ttcggtagtggagggaagagacacggagaggaatgtgggttttgtgtgcacgaattagcttagctagcttagaaa*tgt*
*gtgtgtgtgtgtgtgtgtgtgtgtgcgtgtgtgtgtg*ctgtgtctctgctggtgctgcgcagtgtgctttgtgtttgc
agtgaatcattgtgtgttttcaggaagctgtgtggatgagtaagccatgaaaagaggtgggggggggggggggggagg
cggggtgggggggctttccaatacacagacccctccctagaaattggattggctgccaagaaatgtcccaaatgtccta
ccttttggaatcagcttttcgttcatatactcatgcttttaaatcaggatcgtaagtatgtaacctcagtgaacaagc
atcaaattcatcctatgcgatttcaccgagggggtttatgtgagtgcagggtcattacaagtgaaacgtcaaatagtt
catctgtttttttgactaatgaatcaaccactctggtcttcatttctctagttgtatcagttgtatgcagaatacatgt
cctcatgatgaaatataaagtcaacaaattctctttgctccaaaaaactagatgactttaggtttccagaagtaaact
ccttattcacttttctagaccgttctctttgaaagcagttgaggcaaagcgtacaacagattctagctcaaagttaac
tacacaattggaggttcatttctgctagaaagaaccaatcacaggtgttgattgtatcttgtgccaatcagcagtgtg
cggtgctgctggcgacgagtgcaagctaaagaaaactgattttacaacctgcagttgaaatgaaatcaggtttgaagg
ttctgggattctcaattttgggagaattcagccactgtgaaacaggttcaattctcccccgctctgatttgtgtcggg
cttcgtctccatggcaacagcagccgaggctcgtggggcattgaagtattctgagccaactccttgacgtaactagac
ttgtgcaacttgcgggacgccagaagtttccaaaatccatggaattataaaacagaaaataacaagaccctgaaaatg
tgtgttgtatttcagagccccggatgccgaacggagtggagcatcacttcagagcctcccggtgaactcacacacgca
gacacactcctgcgtaaaagccctccgcaggagaca
ATGAGAGGTTACCTGCTAACTGCGATCTTCCTGCTGCCCTTG

ENSGACT00000007161

tcatgctccgggccacaacagctgtgaaagcctgaccagatcaaaaaacgcccccagtgtccacaagctaacatgaag
actttttctcatcagtgcttcagcattgtaggtgtgattagtaaaaaaaaaaaccctctgtcctctgctcccacttatt
cctaagagaaacaattcctcttctcccgagaggaattgtcttctcttaacacaaacaatccgcacattccgaagcaaa
ccattgtccatctccttgtctcagcctccgcttctgtccgccgtcctttttccgctcctctttcttttattcgccacc
gtgtcttgggtgaaactttgcacgggatgtctattaatgccatcacgaagccaacatgctcgatcccgctcgggtgag
catgtgcgctctctccagacggagcatccaacgttacacacactcaagagctgacagtgttcaacatgaaagccccac
acacactcacacacataaacacattcctgtatttctatctttgtgaggggagccttgctaacattatgtattcccctg
ctcaatacccaaacctgaacttggcctccacttggctccaaccctcaaccctaaacccccctgcccttaatcctcagcc
ctcaacccttaatcctcagacctcaacccttaatcctcaacccttaaccctcaagcctcaacgctcaacccttaaccc
tcaaccctcaacgctcattccagaggttcccaaacctttccgtcacggccccctttgagaacatagagagtgaaacta
cacaacacactagatgttgttaaaggcagaatgattacacaggatttcttttaaaaaggatagaaagactgaaaacga
ggccacgcccccaagaccctaataggtccaaatgtcttgcagcatgtcagtcggttttagtaattcaatcaatagtaa
tgatttgaacttcagaacaatattaaatattaaaatcatctgcttaatgaatttggatcattgtaagcctgtaaacca
acaacagtcaaaccagtaataaactgcatttgatggattttcacagtatgagaagcttgttaactacaccaccaatct
ctgaagatttgcactcttatctcctattcactaaattgaaacatcactgaacagtaccaaacaaaaagccagtttaat
tgggttaactcaacctttttattagctagtgtgttaaataatttctttttttcatattaaaagatgtctttgtagtaaaa
aaattctagatttacaactttactgatcgctgttctgttcactcacaccacataactgaccagaggaacctcgtgcgt
ttataacgtcacatcccttctcgctatggaatgtgcggcgagtgtgctgaacggaaagctaatgtggccgtacattga
gggtccaactgtaaatgttaaatcacttattgtagacaataagtgcttcacttatagttcatctatttctcttcttga
tctcttgg*cacacgcacacagacacacacacacgcacacacacactcaca*ccgatgaagttctgactgcagggtgagc
taccatcagtgtctttatgtggggtatcttttcttttccttagaaatgaattagtggcagtttgtgctcgagcactcc

tcatgaaagctttcctacccaatggcatcagcacccattatggtggccttggcagagctgcagactctcattcacgtc
gtgtcttgtgatttttcttctttttgtgtgtttgcctgtgtgtgtccttctgactcattctagcaaaaatatgaatg
aaggtcctgtctgtgtgggctggtaaggcattaatattcagcatgcaggactcaaatcaaatgtgtcccgtttggatg
tagaaatgtgtatttttctgtagcagagtttaaattcaataaaggttcaatgtctgccatcatttaaaaaccaaagtg
catgataacatgtgaatctatttacagattgagcagaagatggagcagaaggaagtttaggtccacgtggttcagttc
agatccaaactcagcagctcgctcagctgttgctcctgacaacggcagggggaggggcttgctgctggcagggtttcg
gtaaagtaggaatgccaagctctccctctgggacgaggcctgactgctcagtcctgctacttggctttactcatgtga
acccccccctccccccgggctcgatgggatccatgcacatgggctcattgcagacatgcggttttcaaaaatgacctcc
agtccaaacgtttggctgcaactactgaattcctttatctgcccttttattcactaccatgtcaaatagcattctccg
aacccagcaggacatgtttaaacccaaccaagggtccaaaatgcacatatttatttcgctataagaggaagaatcagc
aaaataaatcatcatgtttgacgagctggaagtaggataaccttttttttttctggttagtgaattgtttgcaggatat
ccccaaaaacgttaatgggaaatacaagagggccaggcaacaagggggaatttacagaatgctttgttatcattttta
ttctttatccatttcatctcatgagcatattcaacattttaaacctgcatttcgcacaagtgtgctgtttgaagcatc
ttaaaaacgatcactaaaataaggcttaagagcaaagtagaaggtaaactaaagagctcaacacagacagacccagat
ataaatggagattttataaacatcacggttaaaacatccccagagtgctcttatggttgttgagatttatatacacac
ttgaaaaaagcttttacgaaagatactctataatgtgatatttatgtcctggtccgacaatatggagacacaatgtgg
acacagtgtcttgatgtcccacactcttgtgtttttcaggtgtgaaggtcagctgacgtcctgttttgtccggctctc
acaactttatgttgggaagaaactaagaaaagcaac
ATGTGTTTAACTAACGTCAACCCGCTGATCCTCACCCTCCTCCTCCTCCTCTTCTTACCCTCG

MEDAKA PAC$_1$s

ENSORLT00000022251

(…) nnnnnnnnnnnnnnnnnnnnnnnnnnnnnnnnnnnnnnnnnnnnnnnnnnnnnnnnnnnnnnnnnnnnnnnnnnnnnn
nnnnnnnnnnnnnnnnnnnnnnnnnnnnnnnnnnnnnnnnacagcaggactcatccatctagcgaggatgtggattggtc
agaaatggtgctctgtatgtcttctctaccaaaatatttctaaaacttttgggtttttaacattttgaaagaaccttt
atggtgcccaattggggccaatagttttgaaaatcaaaaatacaacaatttaaaactaaagctgaaaaactggcgttg
aaactcaaaataaaaaacaattaaaaaaaaaactgacagctcccaccctcctcattctgattggtccttaatctaagc
cctgccccaactcgccagaacggggccaaaagtcgaaactgaagatttttgattcaattttttttaaaaaaagttgaa
agtgaaaaaaagatttgaaaggaaaatctgagtttgtgaatttcaaatctgaaaaattgaaactgaaactaaacaagg
ttatatttgacttacgataatttacgtttttaaagtcttcttttcattttattcattttgagtttcaattttcagcaa
ataatgagccaaactttttggtggcatacgttcaccctattctacatcccagtcatgacctgaatgcgtcatagagt
ttttattagcatgatactatagaaataacatatatagaaagaaggaagggactcatataacacagatatgaatttatt
tctatccaattatttgtgcattgctttgctcggagaatttagcggctgctgacttgtttacctgctgcttagcaacag
agatgctctgaggatgacaggcttttcctcaaaacagaaccagcttcaagaaatatgactcctgtcaagaagaaaaac
ggaggcgatgtttcgctgttgacagaaactgatggcgtttgttgaacgagctgaagggagtattgtcaggaagaggct
gtgtaggtttgagacggctccggtgacgctccatagcatccgatcggcatccgatccgctctcatctgcaatccgatg
aagagaggaagtgaatccgagggcatcttcacagtctgctgaccataactaggcactgaggcctgaagtgaaatcata
ccaggaggcagagtgatgagtcagctgtggctcagagggctggactgtattacacctaacatttctgtatgtgagcaa
aggggacgcggtgaggaggctgagcggagactgggatgaggagaaggagggaacatgagcagagactgcaataccttt
agggaaatcccagatgcctgtactctcatgtatttgggttcctaaggctttagtggtgtattgtgtggaacataaaat
ggtgaatgactggaggaagcagtttggagcagatgagagatcaaaacatggacttaatgaaaatatttgcccccacca
tcatgccacaattaacgttccaaaacacacacatagg*tgtgtgtgtgtgtgtg*tagcctttccaactcttgaattgac
taattgtaatggacaccattctgtcctgcagtcgtcctggactaaaagaccagatcgctttcagaattcctactctgt
ctttgagcaaaaatgtcatcaccctcgccaccaattaaaaaactcatccagtttgcccacacctactacctgggcaaa
aggaattatgggcatggtgaccnnnnnnnnnntaggaagaggcagccatgttgaattaaaaaaaaaatcctttctagt
accagctacaagttacaacgccgcttttatcacctcttaactcctcgagcatcattgtgagccacttcaatccatgat
ccgctggctccacctgtatgaaacaacataacaaatgacatacacacattgggaatgtttttgttaacaaaaaacatc
agttgccaaggaaatccaaaaatgtacttttgctggtttacctaaaactacacagacctgttcatgacccaaaggcaa
ccaaaaacttaaatggttgctatggaaataaaccaaacgcagctctgaccaggctggaggagagaggggcaacgttga
gtgatgggcctgaagtagctgctcagccagtgagggcggttaaaaaggggtaatgg
ATGGGCGGTGAGGGCCGTACAACACTTCTCACGCTTAAACTCAAG

ENSORLP00000016315

gagaacatatttttgtgtgatggcttaagggactttaaccgggctttaagcgcagattagcggagacaaattctgtgg
agccacaaatcatgcttctccatctgttaggggtgtgaccaataattgacttaatcgataaagcaacgtatattccta
aaatccaactagatccatctgtctgaagtaagtttttaatcaaattgcccaactctttataaaatttcaaaagtcgat
tataatcaaaattggaaaataccatttggattgaggcattataggtttattttactatagcataaaaacgaccaaggg
tcatcaagggtcatcatcagttcaatcctaaaggattaaaaatagactattagtgtgcatcccagatagtaaaaaagg
ggattttagtttttttgagcaatacaacaactagaagagcaaagtttggaactgccaaagtctttcaaatgttttgtta
gcaaccagttgatggtttgctcctgtgctcagtgtaacatctattagtcatgatagtacagcagttacaagaatcaat
acagttgtatctttgtgtgaagccaatcctcctgggctttctcaatttgaactattgacattcagtgaagtcattcaa
gatagatctaatataattatggttcctttaactgaagcttacgatcatgacatcagagataaaagcagatgagttgta
aagaaataatatgtctttttagataacagactagctcatatttttttatttggcgataagtattctaaaaaaaaaaaag
ataaaaggaacaaaacgagcaggataagtaaacagcaaagtagatgtggcagggggtaaatttaaaaaagttaagaaa
ataacagaaaattacaaaaacatttatttcatctcttaaatgggggaaaaccaactagttttagacataacaagttt
tataagtcttgcaaacatttgtccaacaaatgtcgagattgtcaaattttgaataaatctgagttccatccatccatg
ttttatttgttattatgaatctccagtggattctgtccacagctgataaaagaaaatgctttgagttcacaattaaac
tcacattctgtcatgactgttgtctgtggggcttcattgtctgaaccaagtggctgagatgaagtatgtttgtataat
ttccccattgatccatgaagttgtcttctttaagaccaatgagatgaggtcagagacaataaagtgagaaggctgaga

gaagtgcatctattcttctaattaacagacattcatctcgtttatgatagatttaaagacccccatctaacatttctc
gtcagtttttctttgtttttatgctgcagtcattcatttattcattcattcattttctgtaccgctttgtccctttcg
gggtcacggggctgccggagcctatcccggctacttgggcgtaggcaggggatgccctggacaggtcgccagtctgtc
gcagggtagaacgtccacaatcacacacccatgcactctcacactcacacctatggacaatttagagttgccaattaa
cctatgaagcatgtttttggacagtgggaggaagccggagatcccggagagaacccacgcatacacggggagaacatg
caaactcctatgctgcagtcatttgataaaattttgttcttttttttaagatttaaattattcaaattagaagaaga
gaacccgtagattccgaatggtactgaacagctttcaggaggaagccaactctcattaagtcctgtgttaaattgaat
aaaaaacgcatctacagacattatttttaaatcaagagttttgattttattggactgcaaagttcagctgcatggtgg
tgaagtgggtagcgctctcgcctcactgtgacgaggcactggtttgaatctttctttgtacagtttgcgtgttctcct
catgcatgcgtggtttttttttctgggcactccggcttcctaccacagtcagaaaacaggcttcataggttgactggtt
gactgacccttaagtgtgcatgtgtgagtggagtgcaggagcgattgtttgccctgccacagaccggcaacctgtcca
gggtgtaccctgccttggcccagcggtagctaggacatgctctggcaacccttgaccccgatagggatacattgggtt
aagaaaatggattgatggatagagtttaaacctattgcatgattaaagtgttccagggtttttttttttttgatcggct
ggtgccattcccatggatcccaatggactgaaatatctcacactttatcttttcttgcccaatgccccatttttagtg
tttttgaactgagtggagtaaactccagtaagtgtgtctaaccatttcatggcaagcttttgccgttcaggatcacac
tggtgacgtcaaagattactttttgaaaacactgaccattacttcaggaaaactttagaaccccttccccaaatccca
ccatcgacaaaccaaagtgaccatatggggcttttgtgaaaaaagaatttgacatcagtgaaggccactcatgggcga
taaagtatcaagctggataagccaagttcaatgttctgtccggaggctgcagcggattctgctgtcttcttttcagac
agagagcagtttagaaatcatccagaattgtggaaacactcactcagcattgagcctcccactgagaaagtgcttcca
tccatcaactgaatcaattgtgaagcagagtgactatgatggatgctcccccagctgtgactgatcatatcaccatcc
taatcctcatgcccctcctttgtgatctgtcaccctatttgttgtgccacttttcctttttttcctactgttgggaaa
gataatgtctgaaagccttttctgtttgggttcaatcagagaaatgaccttcctttgggtcgctgagcatggggatgc
cagcagctctgcagagctgcaggcagagctttgagtcgttcattc
ATGATGACACTTTCTCTGGTTTGTTTCCTCTTGCAGGGTTCAAATCAGCAGGTGCCCTCCAATTGCGTGATCAAGAGG
GAGCAGGAGAAATGCATGGAGATGATGGCGCTGCACGATCCCAA
